# Editorial: Deep brain stimulation think tank: updates in neurotechnology and neuromodulation, volume VI

**DOI:** 10.3389/fnhum.2026.2007514

**Published:** 2026-10-05

**Authors:** Chance R. Fleeting, Aysegul Gunduz, Michael S. Okun, Joshua K. Wong

**Affiliations:** 1 University of Florida J Crayton Pruitt Family Department of Biomedical Engineering, Gainesville, FL, United States; 2 Department of Neurology, College of Medicine, University of Florida, Gainesville, FL, United States

**Keywords:** deep brain stimulation, electrophysiology, movement disorders, neuroimaging, neuromodulation, neurotechnology and brain-machine interface

Over the last few years, the landscape of deep brain stimulation (DBS) and neuromodulation in general has undergone a profound evolution, rapidly transitioning from static, open-loop therapies to dynamic interventions informed by biomarkers and our ever-expanding understanding of human pathophysiology. With an estimated 300,000 devices implanted globally for neurological and neuropsychiatric conditions, the field is rapidly advancing beyond traditional management toward more comprehensive, patient-specific approaches. Since 2012, the DBS Think Tank has met annually as an open forum to track these advancements, highlight the most recent breakthroughs, and discuss the future of neuromodulation by bringing together the world leaders in neuromedicine and neurotechnology.

This year, DBS Think Tank XIII hosted a broad array of topics, including neuromodulation for gait, brain networks, neuroethics, interventional psychiatry, closing the loop of device development, and how genetics and neuromodulation can be integrated. As with prior iterations, the proceedings for DBS Think Tank XIII (Fleeting et al.) have been published to support our goal of making this material widely available to neuromodulation clinicians and researchers worldwide. Accompanying this annual report, the *Deep brain stimulation think tank: updates in neurotechnology and neuromodulation Research* Topic has been developed with the support of the Frontiers Editorial Office to further this goal and capture the latest advances in neuromodulation as they occur.

In this editorial, we showcase and summarize the articles included in the sixth volume of this Research Topic that together cover the following topics: (1) new approaches to clinical translation, (2) evaluation of different surgical methodologies, and (3) case studies of novel patient-centered neuromodulation paradigms.

## Engaging all stakeholders in neuromodulation

Translating advanced neuromodulation into clinical practice has been a notorious rate-limiting step, requiring rigorous attention and oversight. Johnson et al. presented a framework for DBS clinical trials aimed at accelerating patient recruitment, improving retention, and streamlining this critical step by proposing strategies to enhance public education, optimize patient-centered study designs, engage participants as equal stakeholders, and reduce the logistical, financial, and technological burdens placed on trial participants. This complements the work of King et al., which carried this goal of translation forward by investigating clinicians' views on using a patient decision aid for DBS in Parkinson's disease, thereby highlighting the need for structured tools to guide complex treatment choices.

## Surgical techniques and optimization

Continuous advancements in neuroimaging, robotics, and data analytics are refining the physical implementation of DBS. Meng L. et al. conducted a retrospective study comparing the efficacy and safety of robot-assisted surgery against traditional DBS implantation, finding that the robot-assisted approach using the Sinovation system yielded a significantly lower average targeting error of 1.43 ± 0.51 mm compared to 1.91 ± 1.38 mm for the stereotactic framework. Additionally, the robot-assisted approach resulted in significantly greater reductions in both UPDRS-III motor scores and levodopa equivalent doses at 3, 6, and 12 months post-operatively.

To enhance targeting precision, Patriat et al. introduced “PICS,” a novel patient-specific landmark within the posterior limb of the internal capsule signal designated to improve surgical targeting of the thalamic nuclei for neuromodulation. Building upon this, Mikroulis et al. detailed an interpretable, patient-specific approach to DBS optimization using MRI and clinical review data to tailor stimulation parameters. This approach leverages lead reconstruction data and Volume of Tissue Activated (VTA) simulations to estimate optimal contact positions and suggest appropriate stimulation. By evaluating electric field overlaps, the tool also allows for the integration of prior clinical evaluations to fine-tune settings and avoid adverse effects while minimizing computational overhead.

## Novel neuromodulation paradigms

Remaining an essential tool by which we uncover specific electrophysiological applications and troubleshoot complex clinical responses, granular case studies hold a distinct position within early discovery in neuromodulation. In both Eisel et al. and Meng D. et al., case studies detail new and interesting applications of neuromodulation. In Eisel et al., target-specific modulation of cortical narrowband gamma by basal ganglia nuclei was explored in a patient with Parkinson's disease, revealing that therapeutic stimulation of the subthalamic nucleus and globus pallidus internus successfully suppressed narrow-band gamma activity in the primary motor cortex. This suppression accompanied clinical improvements in rigidity and dystonia, whereas non-therapeutic stimulation of the globus pallidus externus failed to modulate this activity or provide clinical benefit. In a separate case, Meng D. et al. document the effective management of a patient with Meige Syndrome experiencing subthalamic stimulation-induced dyskinesia, successfully resolving adverse effects through the timed alternation of different contacts.

## Conclusion

The studies presented in this volume provide a survey of the recent innovations, challenges, and opportunities within our field, detailing “the evolving landscape” of neuromodulation—the central theme of DBS Think Tank XIII. Iterative advancements in neuroimaging, surgical methods, and patient-centered care build upon the current technology year after year, furthering our understanding of individualized neuromodulation therapy. With our overarching goals remaining the same, each of these contributions helps translate cutting-edge technologies into increasingly more efficient and effective forms of therapy. Ongoing multidisciplinary collaboration among clinicians, researchers, and engineers, as fostered by DBS Think Tank, is essential to advancing as a field.

